# Flavoenzyme-mediated reduction reactions and antitumor activity of nitrogen-containing tetracyclic ortho-quinone compounds and their nitrated derivatives

**DOI:** 10.17179/excli2017-273

**Published:** 2017-05-11

**Authors:** Milda Peciukaityte-Alksne, Jonas Šarlauskas, Lina Miseviciene, Audrone Maroziene, Narimantas Cenas, Kastis Krikštopaitis, Zita Staniulyte, Žilvinas Anusevicius

**Affiliations:** 1 Institute of Biochemistry, Life Sciences Center, Vilnius University, Sauletekio av. 7, Vilnius, LT-10257, Lithuania

**Keywords:** heterocycle quinones, ortho-quinones, enzymatic reactivity, antitumor activity, apoptosis, DFT computation

## Abstract

Nitrogen-based tetracyclic ortho-quinones (naphtho[1'2':4.5]imidazo[1,2-a]pyridine-5,6-diones, NPDOs) and their nitro-substituted derivatives (nitro-(P)NPDOs) were obtained by condensation of substituted 2,3-dichloro-1,4-naphthoquinones with 2-amino-pyridine and -pyrimidine and nitration at an elevated temperature. The structural features of the compounds as well as their global and regional electrophilic potency were characterized by means of DFT computation. The compounds were highly reactive substrates of single- and two-electron (hydride) - transferring P-450R (CPR; EC 1.6.2.4) and NQO-1 (DTD; EC 1.6.99.2), respectively, concomitantly producing reactive oxygen species. Their catalytic efficiency defined in terms of the apparent second-order rate constant (k_cat_/K_M (Q)_) values in P-450R- and NQO-1-mediated reactions varied in the range of 3-6 × 10^7^ M^-1^ s^-1^ and 1.6-7.4 × 10^8^ M^-1^ s^-1^, respectively. The cytotoxic activities of the compounds on tumor cell lines followed the concentration-dependent manner exhibiting relatively high cytotoxic potency against breast cancer MCF-7, with CL_50_ values of 0.08-2.02 µM L^-1^ and lower potency against lung cancer A-549 (CL_50_ = 0.28-7.66 µM L^-1^). 3-nitro-pyrimidino-NPDO quinone was the most active compound against MCF-7 with CL_50_ of 0.08 ± 0.01 µM L^-1^ (0.02 µg mL^-1^)) which was followed by 3-nitro-NPDO with CL_50_ of 0.12 ± 0.03 µM L^-1^ (0.035 µg mL^-1^)) and 0.28 ± 0.08 µM L^-1^ (0.08 µg mL^-1^) on A-549 and MCF-7 cells, respectively, while 1- and 4-nitro-quinoidals produced the least cytotoxic effects. Tumor cells quantified by AO/EB staining showed that the cell death induced by the compounds occurs primarily through apoptosis.

## Introduction

Quinones constitute an important class of naturally occurring compounds which play a pivotal role in the diverse biological systems (Nohl et al., 1986[36]; Monks et al., 1992[34]; Bolton et al., 2000[8]; Monks and Jones, 2002[35]). They also represent a large class of redox active xenobiotics which may induce a variety of hazardous effects including cardiotoxicity, neurotoxicity, immunotoxicity and carcinogenesis (Bolton et al., 2000[8]; Monks and Jones, 2002[35]; Bolton and Dunlap, 2017[7], and refs. therein). On the other hand, quinoid compounds are widely used for treatment of viral and microbial infections, and comprise one of the major classes of chemotherapeutic agents (Riffel et al., 2002[42]; Asche, 2005[2]; Garuti et al., 2007[22], and refs. therein). Owing to electrophilic (electron-accepting) capacity of quinones, one of the primary stages of their (cyto)toxic and/or therapeutic action is related to their bioreductive activation mediated by oxidoreductases producing semiquinone radical and/or hydroquinone (diol) species (Rooseboom et al., 2004[44]; Siegel et al., 2012[50]). The single-electron reduction of quinones is typically catalyzed by single-electron transferring flavoenzymes such as NADPH-cytochrome P-450 reductase (P-450R, CPR), NADH-cytochrome b5 reductase (CB5R), NADPH:ubiquinone oxidoreductase (complex I), etc. (Bachur et al., 1979[3]; Ross et al., 1996[45]; Čėnas et al., 1994[13]; Matsuda et al., 2000[32]; Rooseboom et al., 2004[44], and refs. therein). The semiquinone radicals formed are readily back-oxidized by molecular oxygen (the so- called futile redox cycling) generating superoxide along with other reactive oxygen species (ROS) (Powis, 1989[40]; Rooseboom et al., 2004[44]). The obligatory two-electron (hydride) reduction of quinones to their diol forms, typically mediated by two-electron transferring flavoenzyme NAD(P)H: quinone acceptor oxidoreductase (DT-diaphorase (DTD), NQO-1), affords the protection against cytotoxic effects of quinones diverting them from their redox cycling (Joseph et al., 2000[25]). NQO-1 is well-known for being a major xenobiotic metabolizing enzyme, whose induction is frequently associated with the protection against toxic and/or carcinogenic effects of a large variety of electrophiles. On the other hand, the two-electron reduction of certain structure quinoid xenobiotics and drug agents to unstable diol species is accompanied by their chemical rearrangement to highly reactive intermediates and/or spontaneous auto-oxidation generating ROS (Gutierrez, 2000[24]; Beall and Winski, 2000[4]; Colucci et al., 2008[15]; Rooseboom et al., 2004[44]; Siegel et al., 2012[50]; Bian et al., 2014[5], 2015[6]). Because of the enhanced expression of NQO-1 in tumors and tumor cells, this enzyme has become one of the most attractive targets for bioreductive activation of various redox active xenobiotics and drug agents including quinoid-based compounds (Danson et al., 2004[18]; Rooseboom et al., 2004[44], Siegel et al., 2012[50]; Bian et al., 2014[5], 2015[6] and refs. therein). The quinone-mediated enhanced generation of ROS was proved to contribute to the "non-specific" cellular damage and/or induction of specific ROS-sensing signaling pathways thus causing cell cycle arrest, senescence, apoptosis and other events (Mates and Sanchez-Jimenez, 2000[31]; Liou and Storz, 2010[29], and refs. therein). 

Among a broad variety of quinoid-based compounds, the natural and synthetic heterocyclic quinones were shown to be efficient enzymatic substrates and potential cytotoxic/anti-tumor agents (Pan et al., 1993[38]; Rooseboom et al., 2004[44]; Deegan et al., 2006[19]; Colucci et al., 2008[15]; Garuti et al., 2007[22]; Valderrama et al., 2008[55]; Chen and Hu 2009[14]; Vásquez et al., 2010[56]; Koyama et al., 2010[28], 2011; Brandy et al., 2012[10]; Delgrado et al., 2012[20]; Zhang et al., 2012[60]; Bian et al., 2014[5], 2015[6]; Cai et al., 2016[11]). Compared to the heterocyclic para-quinoidals, the enzymatic reactivity and/or cytotoxic/antitumor activity studies of ortho-quinoid compounds are still limited to a few groups of the natural and synthetic ortho-quinoid compounds such as 9,10-phenanthroline-5,6-dione (phendione, PD) (Deegan et al., 2006[19]), pyrroloquinoline quinone (PQQ) (Shen et al., 2009[49]; Shankar et al., 2010[48]), tetrahydropyran-fused ortho-napthoquinone (β-lapachone) and its ortho-quinone analogues (Bian et al., 2014[5], 2015[6], and refs. therein), tetrahydrophenanthrofuran-diones (tanshinones) (Zhang et al., 2012[60]; Dang et al., 2011[17]; Cai et al., 2016[11], and refs. therein). The ortho-quinoid compounds have proved to be in general more effective electrophilic and redox active agents than para-quinoid compounds due to the enhanced electrophilic character of the vicinal carbonyl moieties of the former compounds (Tonholo et al., 1998[54]; Campodonico et al., 2009[12]; Borges et al., 2014[9]). 

**Figure 1:**
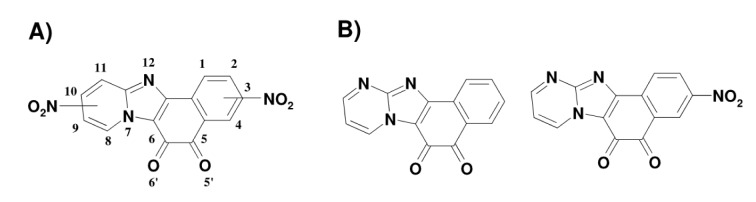
Chemical structures of N-tetracyclic ortho-quinoidals and their nitrated derivatives used in this work: A) NPDOs and B) pyrimidino-NPDOs (PNPDOs)

Herein we report the study of enzymatic reactivity and antitumor activity of the synthesized fused N-containing tetracyclic *ortho*-quinoid compounds (naphtho[1'2':4.5] imidazo[1,2-a]pyridine-5,6-diones, (P)NPDOs) and their nitrated derivatives. The chemical structures of the compounds are given in Figure 1(Fig. 1) (for NPDO compounds, the numbers denote the relative positions of the nitro groups). Their structural features as well as the global and the regional (fractional) electrophilic potencies were characterized by means of DFT computation. Their enzymatic reduction reactions and redox cycling ability were assessed applying single-electron transferring P-450R (CPR) and two-electron-transferring NQO-1 (DTD) as the main flavoenzymes being responsible for bioreductive activation of a large variety of redox active xenobiotics including nitroaromate- and quinoid-based compounds. The cytotoxic activities of (P)NPDO and nitro-(P)NPDO quinoidals were estimated against the human A-549, MCF-7 and HL-60 tumor cell lines. 

## Materials and Methods

### General

All chemical reagents and solvents were obtained from commercial suppliers (Sigma-Aldrich (St. Louis, MO, USA), TCI-Europe (Zwijdrecht, Belgium) and Merck (Darmstadt, Germany)). The purity of the compounds was checked by TLC (silica gel 60 F254 aluminium plates (Merck, Darmstadt, Germany)) and visualized by UV light. The flash column chromatography was performed by silica gel Wakogel C-200 (Wako Chemical, Osaka, Japan). The melting points were defined in open capillaries by using MEL-TEMP equipment (Barnstead Thermolyne Corp, Dubuque, IA, USA). UV-VIS spectra were obtained by Lambda 25 UV-VIS (Perkin-Elmer, Waltham, MA, USA) spectrophotometer. IR spectra were recorded in KBr on a Perkin-Elmer spectrophotometer (FT-IR Spectrum BX II). NMR spectra were obtained with Bruker spectrometer (^1^H - 400 MHz and ^13^C - 100 MHz) in d-chloroform or d_6_-DMSO by using residual solvent signal as an internal standard. HRMS spectra were recorded by Dual-ESI Q-TOF 6520 (Agilent Technologies, Santa Clara, CA, USA) mass spectrometer.

### Synthesis 

**Figure 2:**
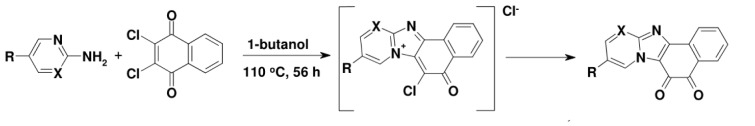
The condensation of 2,3-dichloro-1,4-naphthoquinone with 2-amino-N-heterocycles to obtain NPDO quinoids ( X=CH, R=H), 9-nitro-NPDO ( X=CH, R= NO_2_) and pyrimidine-NPDO (X=N, R=H) compounds

Naphtho[1',2':4,5]imidazo[1,2-a]pyridine-5,6-dione (NPDO**), **9-nitro-NPDO**, **and naphtho[1',2':4,5]imidazo[1,2-a]pyrimidine-5,6-dione (PNPDO**)** were synthesized by condensation of 2,3-dichloro-1,4-naphthoquinone (0.01 M) with 2-amino-N-heterocycles (0.02 M) in dry 2-butanol (300 mM) (Figure 2(Fig. 2)). The reaction mixture was stirred under argon and heated at 100 °C in a closed vessel for 36 h (for synthesis of 9-nitro-NPDO, the time was increased to 96 h). The reaction mixture was concentrated, allowed to cool to 10 °C, filtered and washed with cold 1-propanol (3x20 ml), hexane, and dried. The resultant products were re-crystallized from 1,2-dichlorobenzene or purified by flash chromatography (CH_3_OH/CH_2_Cl_2_ (1/10)).

*3-Nitro-naphtho[1',2':4,5]imidazo[1,2-a] pyridine-5,6-dione (3-nitro-NPDO), and 3-nitro-naphtho[1',2':4,5]imidazo[1,2-a] pyrimidine-5,6-dione (3-nitro-PNPDO****)*** were obtained by the nitration reactions of NPDO and pyrimidino-NPDO (PNPDO) quinoids, applying fuming nitric acid in 98 % H_2_SO_4 _at reflux. 1 g (4 mM) pymd-NPDO was added to the stirred ice-cold mixture of 0.8 ml 95 % HNO_3_ and 25 ml 98 % H_2_SO_4_, heated to 110 °C and refluxed for 3 h, then allowed to cool to room temperature and poured on 200 g of ice. The precipitate was filtered, washed by 5 % sodium hydrocarbonate solution and subsequently by distilled water to the neutral reaction. After drying, the final product, re-crystallized from CH_2_Cl_2_, yielded a small red crystalline powder.

**Figure 3:**
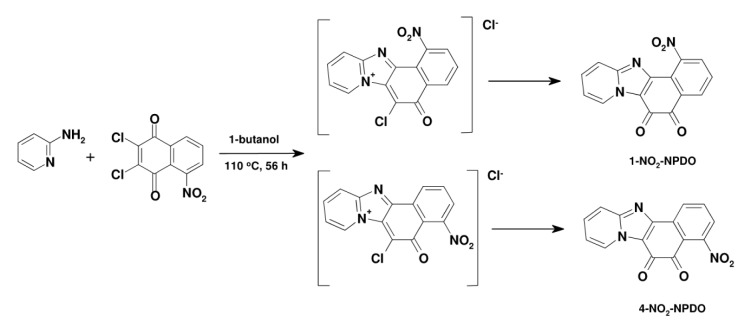
The synthesis of 1- and 4-nitro-NPDOs by condensation reaction of 2-amino-pyridine with 5-nitro-2,3-dichloro-1,4-napthoquinone (110 °C/56 h)

*1- and 4-nitro-naphtho[1',2':4,5] imidazo [1,2-a]pyridine-5,6-diones (1- and 4-nitro-NPDOs) *were synthesized by condensation reaction of 5-nitro-2,3-dichloro-1,4-naphthoquinone (NCNQ) with 2-aminopyridine during the prolonged heating in a polar solvent (Figure 3(Fig. 3)). 2-Aminopyridine (1.88 g, 20 mM) was slowly added to the stirred NCNQ solution (2.27 g, 10 mM) in dry 1-butanol (200 ml). The suspension was stirred under argon and heated at 110 °C for 56 h, then concentrated and allowed to cool to 5 °C. The obtained product was filtered and washed with cold methanol (3x20 ml), then hexane, and dried. The resultant mixture of 1-nitro- and 4-nitro-NPDO isomers was fractionally crystallized from hot chloroform, producing two crystal fractions, which were purified by flash chromatography on SiO_2_ with ethyl acetate. After re-crystallization, the more and less soluble fractions yielded 1-nitro- and 4-nitro-NPDO isomers, respectively, with molar ratio of 1:3.

### Characterization

*Naphtho[1',2':4,5]imidazo[1,2-a]pyridine-5,6-dione (NPDO).* Orange solid. Yield: 43%. Melting point: 304-305 °C. UV-Vis λ_max_ (nm): 216, 242, 261, 283, 295, 396. FT-IR (KBr): ν (cm^-1^) 1687, 1651 (C═O), 1629, 1602, 1498, 1482, 1418, 1326, 1257, 1197, 1151, 1089, 927, 899, 766, 723, 699, 622, 576. ^1^H-NMR (d_6_-DMSO): δ (ppm) 9.25 (d, 1H, H-8, J_H8-H9_=5.00 Hz) 8.12 (d, 1H, H-10, J_H9-H10_=5.00 Hz) 7.70, 7.46 (2m, 4H, H-1, H-2, H-3, H-4) 7.14 (m, 1H, H-9). ^13^C-NMR (CDCl_3_): δ (ppm) 181.82 (1C, C-5) 169.33 (1C, C-6) 155.58 (1C, Cquat) 150.56 (1C, Cquat) 135.74 (1C, C-2) 131.71 (1C, Cquat) 131.32 (1C, C-10) 130.79 (1C, C- Cquat) 130.55 (1C, C-3) 130.00 (1C, C-4) 128.73 (1C, Cquat) 126.72 (1C, C-8) 124.75 (1C, C-1) 118.03 (1C, C-11) 116.65 (1C, C-9). HRMS: λ_max_m/z [M+H]+. 249.0652, (calc.: 249.0657).

*Naphtho[1',2':4,5]imidazo[1,2-a]pyrimidine-5,6-dione (PNPDO)*. Bright yellow solid. Yield: 17 %. Melting point: 345 °C. UV/ Vis λ_max_ (nm): 241, 263, 293. FT-IR spectrum ν (cm^-1^): 3049, 3066, 1700, 1654, 1610, 1540, 1523, 1479, 1423, 1396, 1375, 1347, 1241, 1220, 1186, 1153, 1119, 1073, 1014, 938, 920, 887, 850, 832, 815, 784, 757, 736, 723, 696, 669, 648, 610, 578. ^1^H-NMR (CDCl_3_): δ (ppm) 9.53 (dd, 1H, H-8, J J_H8-H9_=6.41 Hz) 8.88 (d, 1H, H-10, J_H1-H2 _=7.93 Hz) 8.36 (dd, 2H, H-1, H-4, J_H3-H4_=9.16 Hz), 7.75 (m, 2H, H-2, H-3), 7.27 (t, 1H, H-9, J_H8-H9_=J_H9-H10_=6.09 Hz). ^13^C-NMR (CDCl_3_): δ (ppm) 187.68 (1C, C-5), 182.16 (1C, C-6) 177.00 (1C, Cquat) 150.16 (1C, Cquat) 149.40 (1C, Cquat) 144.90 (1C, C-4) 136.06 (1C, C-2) 132.86 (1C, C-10) 130.84 (1C, Cquat) 130.82 (1C, Cquat) 129.17 (1C, C-8) 126.99 (1C, C-1) 124.54 (1C, C-3) 117.40 (1C, C-9). HRMS: (C_14_H_6_N_4_O_4_): [M+H]^+.^:250.0608 (found), 250.0611(calc.).

*3-Nitro-naphtho[1',2':4,5]imidazo[1,2-a]pyridine-5,6-dione (3-nitro-NPDO).* Red solid. Yield: 57 %. Melting point >360 °C. FT-IR spectrum ν (cm^-1^): 1702, 1651 (C═O), 1628, 1609, 1544, 1521 (NO_2_), 1504, 1409, 1348, 1325, 1287, 1245, 1210, 1191, 1153, 1115, 1072, 1010, 942, 910, 736. ^1^H-NMR (DMSO-d_6_): δ (ppm) 9.36 (d, 1H, H-8, J_H8-H9_=7.62 Hz) 8.96 (s, 1H, H-4) 8.57 (d, 1H, H-1, J_H1-H2_= 8.54 Hz) 8.43 (d, 1H, H-2, J_H1-H2_=8.54 Hz) 7.93 (d, 1H; H-11, J_H10-H11_=7.63 Hz) 7.75 (t, 1H, H-10, J_H9-H10_=J_H10-H11_=7.63 Hz) 7.32 (t, 1H, H-9, J_H8-H9 _=J_H9-H10 _=7.63 Hz). HRMS m/z [M+H]^+.^ 294.0505 (calc.: 294.0509).

*9-Nitro-naphtho[1',2':4,5]imidazo[1,2-a]pyridine-5,6-dione (9-nitro-NPDO). *Redish orange solid. Yield: 11 %. M.p. 342-3 °C (with decomposition). FT-IR (KBr): ν (cm^-1^) 1694, 1652 (C=O), 1640, 1599, 1551, 1513, 1413, 1344, 1304, 1270, 1240, 1220, 907, 822. ^1^H-NMR (CDCl_3_): δ (ppm) 9.45 (s, 1H, H-8) 8.17, 7.62 (2m, 4H, H-1, H-2, H-3, H-4) 7.75 (dd, 1H, H-11, J_H10-H11_=9.6 Hz, J_H8-H11_=0.73 Hz) 7.58 (dd, 1H, H-10, J_H10-H11_=9.6 Hz, J_H8-H10_=2.06 Hz). ^13^C-NMR (CDCl_3_): δ (ppm) 182.13 (1C, C-5), 164.04 (1C, C-6), 155.40 (C-quat), 135.6 (1C, C-3) 133.5 (1C, C-4) 131.10 (1C, C-8) 127.8 (1C, C-1) 122.3 (1C, C-11) 114.4 (1C, C-9). HRMS m/z [M+H]^+ ^294.0512 (calc.: 294.0509). 

*1-nitro-naphtho[1',2':4,5]imidazo[1,2-a]pyridine-5,6-dione (1-nitro-NPDO)*. Orange red solid. Yield: 17 %, m.p. 292 °C. UV/ Vis spectrum (nm): 240, 296, 398. FT-IR (cm^-1^): 3080, 1701, 1654, 1601, 1539, 1497, 1445, 1415, 1405, 1377, 1320, 1285, 1244, 1218, 1186, 1145, 1003, 929, 905, 868, 833, 768, 750, 715, 699. ^1^H NMR: (400 MHz, d-chloroform): δ (ppm) 9.34 (d, 1H, H-8, J_H8-H9_=6,43Hz); 8.32 (d. 1H, H-2, J_H2-H3 _=7.85Hz; 7.87 (m. 3H, H-2, H3, H-11); 7.74 (t. 1H, H-10, J_H9-H10_=J_H10-H11_=7.76Hz); 7.54 (d, 1H, H-4, J_H3-H4_=7.95Hz); 7.31 (t, 1H, H-9, J_H8-H9_=J_H9-H10_=6. 24Hz). ^13^C-NMR (100 MHz, d-chloroform) ): δ (ppm) 190.20, 184.52, (2C, C-5, C-6), 175.06 (1C, Cquat), 141.92 (1C, C-1), 133.02 (1C, C-2), 130.67 (1C, C-10), 126,85 (1C, C-8), 125.10 (1C, C-4), 122,36 (1C, C-3), 117.11 (1C, C-11), 116.71 (1C, C-9). HRMS: (C_15_H_7_N_3_O_4_) [M+H]^+.^: 294.0510 (found), 294.0509 (calc.). 

*4-nitro-naphtho[1',2':4,5]imidazo[1,2-a]pyridine-5,6-dione (4-nitro-NPDO)*. Red solid. Yield 21 %, m.p. 323 °C. UV/ Vis spectrum (nm): 241, 264, 297, 400. FT-IR (cm^-1^): 3073, 3038, 1695, 1652, 1602, 1541, 1498, 1474, 1450, 1416, 1371, 1325, 1285, 1256, 1240, 1218, 1185, 1256, 1241, 1218, 1186, 1150, 1110, 1006, 931, 905, 826, 806, 771, 718, 703, 686, 574. HRMS: (C_15_H_7_N_3_O_4_) [M+H]^+.^: 294.0508 (found), 294.0509 (calc.)^ 1^H NMR: (400 MHz, d-chloroform): δ (ppm) 9.29 (d, 1H, H-8, J_H8-H9_=6,40Hz); 8.42 (d. 1H, H-10, J_H10-H11_=J_H9-H10 _=7.94Hz; 7.83 (m. 2H, H-11); 7.74 (t. 1H, H-10, J_H9-H10_=J_H10-H11_=7.64Hz); 7.51 (d, 1H, H-3, J_H3-H4_=7.95Hz); 7.27 (t, 1H, H-9, J_H8-H9_=J_H9-H10_=6.12Hz). ^13^C-NMR (100 MHz, d-chloroform) ): δ (ppm) 187.74, 192.19 (2C, C-5, C-6), 177.02 (1C, Cquat), 144.95 (1C, C-4), 136.12 (1C, C-2), 132.98 (1C, C-10), 129,21 (1C, C-8), 127.13 (1C, C-1), 124,56 (1C, C-3), 118.63 (1C, C-11), 117.41 (1C, C-9). 

3-nitro-naphtho[1',2':4,5]imidazo[1,2-a]pyrimidine-5,6-dione (3-nitro-PNPDO). 

UV/Vis spectrum λ_max_ (nm): 239, 319, 430. FT-IR spectrum (cm^-1^): 3049, 3066, 1702, 1654, 1610, 1540, 1523, 1479, 1423, 1396, 1375, 1347, 1241, 1220, 1186, 1153, 1119, 1073, 1014, 938, 920, 887, 850, 832, 815, 784, 757, 736, 723, 696, 669, 648, 610, 578. ^1^H-NMR (d-chloroform): δ (ppm) 9.58 (dd, 1H, H-8, J J_H8-H9_=6.43 Hz) 8.93 (s, 1H, H-4) 8.36 (dd, 2H, H-1, H-4, J_H1-H2_=9.13 Hz), 7.83 (m, 2H, H-2, H-4), 7.27 (t, 1H, H-9, J_H8-H9_=J_H9-H10_=6.43 Hz). ^13^C-NMR (d-chloroform): δ (ppm) 188.18 (1C, C-5), 183.23 (1C, C-6) 179.40 (1C, Cquat) 153.04 (1C, Cquat) 147.18 (1C, Cquat) 145.98 (1C, C-4) 138.46 (1C, C-2) 133.89 (1C, C-10) 132.54 (1C, Cquat) 131.12 (1C, Cquat) 129.96 (1C, C-8) 127.19 (1C, C-1) 125.23 (1C, C-3) 119.10 (1C, C-9). HRMS: (C_14_H_6_N_4_O_4_): [M+H]^+.^ : 295.0460 (found), 295.0462 (calc.) 

### Computational details

The quantum mechanical computation of the compounds was performed by Spartan 10' (Wavefunction Inc., version 1.1.0, 2011, Irvine, CA, USA) software package. Their geometries were initially refined by the mechanical MMFF94 method and further optimized by the semi-empirical PM6 method. The final reoptimization and computation of the compounds were performed by DFT-B3LYP functional with 6-31+G (d) basis set. The geometries of the compounds were globally optimized without symmetry constraints and their stationary points were confirmed by vibrational frequency analysis. The electron affinity (EA) of the compounds was assessed as the Gibbs' energy difference between their optimized neutral and anion free radical states (at 298.15 K). The cDFT-based global reactivity indices of the compounds were calculated applying their LUMO and HOMO eigenvalues, and their regional electrophilic reactivity was assessed in terms of the electrophilic Fukui function (*f* ^+^) values by using the single-point FMO approach (Contreras et al., 1999[16]) employing the PYTHON scripting language.

### Enzymes and enzymatic assay

NADPH:cytochrome P-450 reductase (CPR; EC 1.6.2.4) was purified by the method described in Pechurskaja et al. (2007[39]), and its concentrations was quantified spectrophotometrically at 455 nm (Δε_456_ = 21.4 mM^-1^ cm^-1^). NAD(P)H: quinone oxidoreductase (DTD, NQO1; EC 1.6.99.2) was purified by the established method (Prochaska, 1988[41]), and its concentration was quantified at 460 nm applying Δε_460_ = 11.0 mM^-1^ cm^-1^. Bovine cytochrome *c*, superoxide dismutase (SOD; EC 1. 15.1.1) and catalase (CAT; EC 1.11.1.6) were obtained from suppliers (Sigma-Aldrich (Saint Louis, MO, USA)). 

The enzymatic reactivity of the compounds was measured spectrophotometrically by Carry-60 spectrophotometer (Agilent Technologies) in 0.1 M K-phosphate buffer (pH 7.0) containing 1 mM EDTA, at 25 °C. The enzymatic rates were defined according to NADPH oxidation at 340 nm (*Δε_340_* = 6.22 mM^−1^ cm^−1^) and corrected for the auto-oxidation of the enzymes in the absence of quinoid substrates (0.11 s^-1^ and 0.05 s^-1^ for CPR and DTD, respectively). Typically, 10-15 concentrations of the compounds were used, and each measurement was performed in triplicate. In separate experiments, quinone-mediated single-electron reduction of 50 µM ferricytochrome *c* was monitored spectrophotometrically at 550 nm (Δε_550_ = 20.0 mM^-1^ cm^-1^), and O_2_ uptake was recorded polarographically applying a Clark electrode (Digital Model 10, Rank Brothers Ltd., Cambridge, UK), at 25^ °^C.

### Assessment of kinetic parameters

The apparent turnover number (k_cat_) and the apparent Michaelis constant (K_M,Q_) values were obtained by using Michaelis-Menten equation of v_0_/[E_0_] (s^-1^) *vs.* [Q], where v_0_, [E_0_] and [Q] denote the enzymatic rate, the total enzyme concentration, and the concentration of the compound, respectively. The apparent second-order rate constant (k_cat_/K_M,Q _) values were calculated applying the re-parameterized form of Michaelis-Menten equation (Kolm et al., 1995[27]; Šarlauskas et al., 2016[47]).

### Tumor cell lines and cell viability assay 

Human pulmonary epithelial A-549 and breast tumor MCF-7 cells were cultured in DMEM media (Life Technologies, USA), containing 10 % (v/v) fetal bovine serum (Life Technologies, USA), 100 U/ml penicillin, and 100 μg/ml streptomycin (Biological Industries, Israel), in a humidified atmosphere with 5 % CO_2_ at 37 °C. The adherent cells were detached by 0.25 % trypsin-EDTA. The target cells, at density of 2 × 10^5^/ml, were seeded into 96-well plates to adhere for 24 h. After reaching 60-80 % confluence, they were treated by the compounds for 24 h. Cell survival was quantified by colorimetric assay based on the conversion of MTT to MTT-formazan product. The stock solutions of the compounds were prepared in DMSO (0.1 % (v/v) in culture media). After the 24 h treatment with the compounds, the cells were washed twice with 0.1 M PBS, and MTT solution (0.2 mg/ml PBS) was added. Following 1 h incubation at 37 °C, the overlying media was removed, and formazan crystals were dissolved by 96 % EtOH. The absorbance density was measured at 570 nm (Varioskan Flash microplate spectrophotometer; Thermo Scientific, USA). At least three independent experiments were performed for each concentration of the compounds and the cell survival was assessed as a percentage in respect to 0.1 % DMSO-treated cells. The CL_50_ values were defined by using a four-parameter logistic equation (SigmaPlot software, Inc., San Jose, CA). 

### Double fluorescence Acridine Orange (AO)/Ethidium Bromide(EB) staining 

The type of cell death was assessed by means of the double AO/EB fluorescence staining. After 24 h treatment by ortho-quinoid compounds at their CL_50_ concentrations, the cells were washed with PBS, stained with AO (100 μg/ml in PBS) and EB (100 μg/ml in PBS) and analysed by fluorescence microscope (Olympus IX51, Japan). 0.1 % DMSO-treated cells served as a negative contol.

## Results and Discussion

### The computational study

The quantum mechanical computation showed that the structures of optimized tetracyclic systems of NPDO and PNPDO quinoids have a planar geometry and the orientation of the nitro groups depends on the positions of their attachment. The nitro groups attached at the 3 and 9 positions of 3- and 9-nitro-quinoids were almost parallel to the tetracyclic plane, pointing to their electron-attracting -inductive and -resonance effects, while the dihedral angles of the nitro groups introduced at the 1 and 4 positions were defined to be almost at the perpendicular orientation (Θ ~89^o^), thus pointing to their electron-attracting inductive effects. Note that the close dihedral angle of the nitro group was defined for 4-nitro-NPDO quinone by means of the X-ray diffraction analysis (Θ ~84^o^) (unpublished data). The twisting of the nitro groups is most likely to be caused by the repulsive interaction of the lone electron pair of the nitrogen atom (N-12) of the tetracyclic system with the oxygen atom of the nitro group attached to position 1 in the 1-nitro-quinoid compound, and by the repulsive interaction of the oxygen atom of quinoid moiety with the oxygen atom of the nitro group added to position 4 in the 4-nitro-quinoid compound. 

The electrophilic (electron-accepting) potencies of the compounds assessed in terms of their *LUMO* energy (*ϵ*_LUMO_) along with the electron affinity (EA) values are listed in Table 1(Tab. 1). This table also provides a set of reactivity indices of the compounds estimated applying the conceptual DFT (cDFT) approach (Geerling et al., 2003[23], and refs. therein). Following this approach, the electrophilicity index (ω), representing the global electrophilic power of molecules, is defined by ω = µ^2^/(2η) = (µ^2^/2)S where µ and η denote the electronic chemical potential and electronic chemical hardness (or softness, S = 1/η), respectively, which were approached in terms of HOMO and LUMO eigenvalues of the compounds, µ = (*ϵ*_LUMO_ + *ϵ*_HOMO_)/2 and η = 1/S = *ϵ*_LUMO_ - *ϵ*_HOMO_. Using this approach, the quantitative relationships between the hydride affinity and the ω index values have been defined for a range of *para-* and *ortho*-quinoids (Campodonico et al., 2009[12]), which shows that this quantity can be applied to predict the ease of the two-electron (hydride)-reduction of quinoid oxidants. 

As shown in Table 1(Tab. 1), the PNPDO, which differs from NPDO by the existence of an additional nitrogen atom in the tetracyclic system (Figure 1(Fig. 1)), possesses a higher electron-accepting potency compared to NPDO quinoidal. The attachment of nitro groups to NPDO and PNPDO compounds substantially increases the electrophilic potency for nitro-quinoidals as reflected in terms of the assessed EA, *ϵ*_LUMO_ and ω index values (Table 1(Tab. 1)), which vary depending on the positions of the nitro groups, i.e, 3-nitro- and 9-nitro-quinoidals exhibited a markedly higher electrophilic potency compared to that of 1- and 4-nitro-quinoidals. For the whole set of the compounds, the approximate order of the single- and two-electron accepting potencies were as follows: 3-nitro-PNPDO > 3-nitro-NPDO ~ 9-nitro-NPDO > 1-nitro-NPDO > 4-nitro-NPDO > PNPDO > NPDO (Table 1(Tab. 1)).

Since, in contrast to the unsubstituted quinoidals, the nitro-quinoid compounds contain two electron-accepting/redox-active sites, namely the quinone moiety and the nitro group, their regional electrophilic potencies were assessed in terms of the electrophilic Fukui function (FF) values grouped over C=O atoms of the quinone moiety ( *f ^+^_q_*) and N and O atoms of the nitro group ( *f ^+^_-nitro_*), which may reflect the propensity to accept nucleophile (an electron and/or an hydride ion) at the initial stage of the redox conversion of the compounds. It should be noted that for the assessment of the FF values, the single point frontier molecular orbital (FMO) approach (Contreras et al., 1999[16]) was used in this study as one of the most appropriate methods for redox active compounds which, in contrast to the frequently used finite-difference approximation methods, yields the positive FF values and obeys the normalization condition (Šarlauskas et al., 2014[46], and references therein). As expected, the highest fraction of the global electrophilic potency of the nitro-quinoidals resides upon the C=O atoms of the quinone moiety (the *f ^+^_q_* values varied in the range of 0.42 (9-nitro-NPDO) - 0.56 (4-nitro-NPDO)) as the most preferential site for the reduction of compounds, while the *f*^+^_-nitro_ values of the nitro groups were assessed to be highly dependent upon their position: the *f*^+^_-nitro_ values of the coplanar (π-conjugative) nitro groups of 3-, 9-nitro-NPDO and 3-nitro-PNPDO compounds (0.100, 0.149 and 0.146, respectively) were much higher than those of the twisted nitro groups of 1- and 4-nitro-quinoidals, with *f*^+^_-nitro_ of 0.003 and 0.004, respectively, implying that the nitro groups of 1- and 4-nitro-quinoidals, compared to 3- and 9-nitro-quinoidals, possess a much lower tendency to undergo reductive conversion.

### Enzymatic reactivity

During NADPH-cytochrome P-450 reductase (CPR)-mediated reactions, the time course of oxidation of NADPH (150 µM) by (P)NPDOs as well as by nitrated quinoidals (5-10 µM) proceeded in a single continuous phase (Figure not shown). The reactions were accompanied by concomitant oxygen uptake and its initial rate was supressed by superoxide dismutase (SOD, 150 µg mL^-1^) by 30-45 %, while the introduction of catalase (CAT, 100 U mL^-1^) showed a marginal inhibiting effect, indicating the quinone/semiquinone redox cycling which yields superoxide. For all the compounds examined, the initial NADPH oxidation rates followed hyperbolic dependence on the varying concentrations of the compounds (Figure not shown), with k_cat_ of 28-35 s^-1^ being close to the previously reported turnover number (~ 25 s^-1^) for FMNH^-^-mediated reduction of ferricytochrome *c *(Čėnas et al., 1994[13]). The catalytic efficiency of the reduction of the compounds, estimated in terms of k_cat_/K_M, Q_ values, varied in the range of 3.1-7.2 × 10^7^ M^-1^ s^-1^ (Table 2(Tab. 2), Reference in Table 2: Šarlauskas et al., 2016[47]) which tentatively increased with an increase in their electrophilic potency (Table 1(Tab. 1)). 

The NQO-1-mediated two-electron (hydride) reduction of the compounds proceeded with k_cat_ of 170 - 350 s^-1^ and k_cat_/K_M,Q_ values equaled to 1.6-7.4 × 10^8 ^M^-1^ s^-1^, which were approximately ten-folds higher than those of CPR-mediated reduction of the compounds (Table 2(Tab. 2)). The k_cat_/K_M_,_Q_ of these quinoidals were defined to be far higher than those of ortho-quinoid compounds such as phenanthrene ortho-quinone (9,10-PQ) (k_cat_/K_M,Q_ of 1× 10^6^ M^-1^ s^-1 ^(Anusevičius et al., 2002[1])), β-lapachone and its synthetic analoques (k_cat_/K_M,Q_ of ~ 1-3 × 10^6^ M^-1^ s^-1^ (Bian et al., 2014[5], 2015[6])). 

Like in CPR-mediated single-electron tranferring reactions, the NQO-1-mediated oxidation of NADPH (200 µM) by unsubstituted as well as nitrated quinoidals (5-10 µM) proceeded in a single continuous phase, far exceeding the concentrations of the compounds (Figure not shown); the reactions were followed by the concomitant consumption of O_2_ with rates close those of NADPH oxidation. The rates of O_2 _uptake were suppressed by the introduction of CAT (by ca. 30-65 %), while SOD showed marginal supressing effects (~3-5 %), clearly indicating the generation of peroxide as a main reaction product. 

Measuring NQO-1-mediated reduction of the compounds in the presence of cytochrome *c* (50 µM) as a terminal single-electron acceptor, the initial rates of its reduction were close to the double rates of NADPH oxidation. In introduction of SOD partially supressed the rates (by ca. 30-50 %), implying the superoxide-mediating reduction of cytochrome *c*. In this reaction system, the superoxide is likely to be formed through the single-electron oxidation of the hydroquinones by cytochrome *c* producing semiquinone radicals and their reoxidation by O_2_. It should be noted that semiquinone radicals can also be directly reoxidized by ferricytochrome *c* as it has previously been reported by Winterbourn (1981[58]). The reoxidation of semiquinones by O_2_ and cytochrome *c* has been defined to follow an "outer-sphere" electron transfer (ET) mechanism governed by the single-electron potential (E^1^_7_) and the self-exchange rate constant (k_EX_) values of the reactive species (Meyer et al., 1983[33]; Tollin et al., 1986[53]; Marcus & Sutin, 1985[30]). The reoxidation of semiquinones by ferricytochrome *c*, whose k_SE_ and E^1^_7_(cyt c^+3^/cyt c^+2^) are equal to 5 × 10^5^ M^-1^ s^-1^ (Dixon et al., 1989[21]) and + 260 mV (Rodkey and Ball, 1950[43]), respectively, is predicted to be much faster than the reoxidation of semiquinones by O_2_, with k_EX_ and E^1^_7_(O_2_/O_2_^.-^) of ~100-450 s^-1^ (Zahir et al., 1988[59]) and -155 mV (Wardman, 1989[57]), respectively. Thus the reduction of ferricytochrome *c* by semiquinone is more likely to be indirectly suppressed by SOD by scavenging the superoxide and thus lowering the concentration of semiquinone radical, owing to its reoxidation by O_2_. 

### Antitumor activity

**Figure 4:**
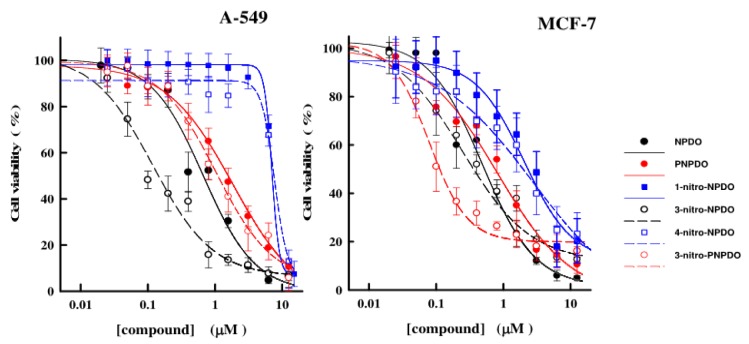
The dependence of viability of A-549 and MCF-7 tumor cells upon the varying concentrations of NPDO and PNPDO quinoidals. The cell survival was assessed by means of MTT assay and expressed as percentages relative to the 0.1 % DMSO -treated cells used as control. Each point represents the mean of the experiments performed in triplicate and the vertical bars the standard error of the mean (S.E.M). The data were analyzed by nonlinear regression analysis by using the logistic type concentration-responce curve.

The *in vitro* antitumor effects of the quinoid compounds against human tumor cell lines, lung carcinoma A-549, breast cancer MCF-7, and leukemia HL-60 cell lines were assessed after their 24-h treatment by varying concentrations of the compounds up to the limits of their solubility (~12.5-15 µM). The viability of A-549 and MCF-7 cell lines versus NPDO quinoidals decreased in a concentration-dependent manner, and the application of four-parametric logistic function provided appropriate fits to the experimental data (Figure 4(Fig. 4)). The estimated CL_50_ values of the compounds, causing a 50 % lethal effect, are given in Table 3(Tab. 3) (Reference in Table 3: Šarlauskas et al., 2016[47]). Unlike A-549 and MCF-7 cells, the cytotoxic potency of NPDO quinoids towards HL-60 leukemic cells was markedly lower (CL_50_ > 15 µM L^-1^), which hampered the establishment of the concentration-response curves and, consequently, the assessment of CL_50_ values (Figure not shown). As shown in Table 3(Tab. 3), the cytotoxic potency of the whole set of the quinoid compounds towards MCF-7 (CL_50_ = 0.08 - 2.74 µM L^-1^) was in general higher than their potency against A-549 cell line, with CL_50_ values of 0.28-7.66 µM L^-1^, except for 3-nitro-NPDO, whose activity against A-549 (CL_50_ = 0.12 ± 0.03 µM L^-1^) was approximately twice as high as that on MCF-7 cell line (CL_50_ = 0.27 ± 0.08 µM L^-1^).

Compared to NPDO quinoidal, PNPDO, possessing a higher electrophilic potency, exhibited lower antitumor activity against both A-549 and MCF-7 cell lines (Table 3(Tab. 3)). The attachment of nitro group at position 3 of the benzene ring of PNPDO quinoidal significantly enhanced the cytotoxic potency of 3-nitro-PNPDO against MCF-7 cell line, with CL_50_ value of 0.076 ± 0.010 µM L^-1^ being 10-folds higher than that of its precursor (CL_50_ = 0.78 ± 0.27 µM L^-1^), while activity of 3-nitro-PNPDO on A-549 cell line (CL_50_ of 1.15 ± 0.48 µM L^-1^) was only slightly higher compared to its precursor (CL_50 _=1.59 ± 0.28 µM L^-1^). Virtually the same was observed for 3-nitro-NPDO quinoidal, whose cytotoxic effects on A-549 cell line (CL_50_ = 0.120 ± 0.03 µM L^-1^) and MCF-7 cells (CL_50_ = 0.280 ± 0.08 µM L^-1^) were approximately 6- and 2-folds higher, respectively, than those of NPDO compound. In contrast to 3-nitro- and 9-nitro-NPDOs, the attachment of the nitro group at the positions 1 and 4 of NPDO benzene ring markedly decreased the cytotoxic potency of 1-nitro- and 4-nitro-quinoidals towards A-549 cell line and to a lesser degree on MCF-7 cell line when compared to NPDO, as evidenced in Figure 4(Fig. 4) and listed in Table 3(Tab. 3). These data show, that, in general, the 3- and 9-nitro substituted quinoids with planar nitro groups and thus higher electrophilic potency for their single- and two-electron (hydride) reduction, as defined in terms of EA, *ϵ*_LUMO_ and ω index values, respectively, exhibited markedly higher antitumor activity as compared to that of the 1- and 4-nitro-quinoidals with non-planar nitro groups and lower electrophilic potency. In contrast to the unsubstituted ortho-quinones, the nitro-quinone compounds bearing a nitro group as an additional electrophilic site may experience more complex metabolic conversion. The (cyto)toxic action of nitro-based xenobiotics and drug agents is frequently related to their reductive activation, i.e, single-electron reduction to the free radicals followed by ROS generation and/or two-/four-electron reduction of the nitro groups yielding highly reactive nitroso-/hydroxylamine intermediates (Tocher, 1997[52]; Knox and Chen, 2004[26]; Chen and Hu, 2009[14], and refs. therein). The position and the geometry of the nitro groups have been reported to be important structural features of the nitro-based xenobiotics in their cytotoxic activity, i.e, the compounds with planar nitro groups have been observed to be more effective cytotoxic agents than their isomers bearing non-planar nitro groups (Onchoke et al., 2004[37]; Takamura-Enya et al., 2006[51]). As reported above, the assessed electrophilic Fukui function values showed that the highest fraction of the electrophilic potency of nitro-quinoidals resides upon the C=O atoms, suggesting that the cytotoxic activity of the compounds is most likely to be due to the redox action of their quinone moiety; however, a relatively high regional electrophilic potency was also defined for the nitro groups of the 3- and 9-nitro-quinoidals, which implies that their nitro groups can be involved in cytotoxic action. 

**Figure 5:**
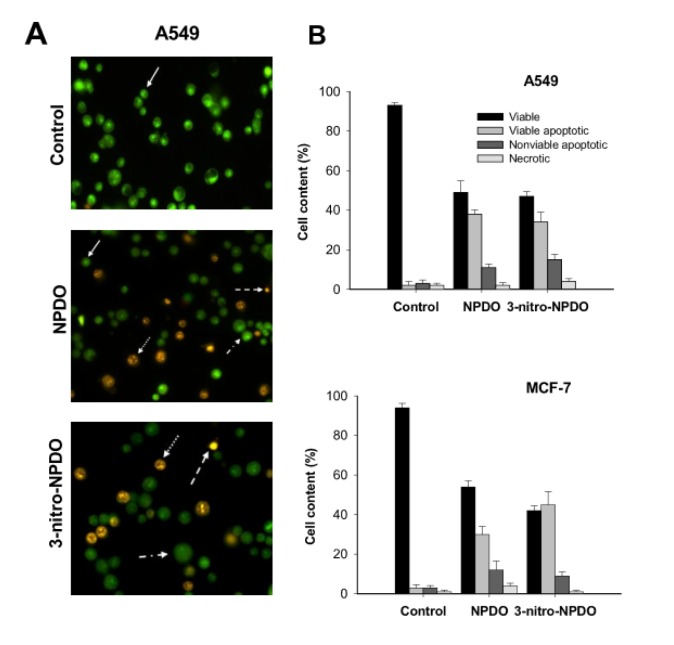
A) Morphological changes of A-54 tumor cells upon 24-h treatment by CL_50_ concentrations of NPDO and 3-nitro-NPDO quinoidals, quantified by means of AO/EB staining (viable cells (solid arrows), viable apoptotic (dashed-dot arrows), nonviable apoptotic (dotted arrows), and necrosis cells (dashed arrows)). B) The percentage of viable, apoptotic and necrotic cells of A-549 and MCF-7 tumor cells upon the treatment by NPDO and 3-nitro-NPDO. Data represent the mean of at least three independent experiments.

To estimate the mode of cell death induced by the ortho-quinoidals, the tumor cells treated with the most active quinoid compounds were quantified applying the dual EB/EO fluorescence staining approach. As shown in Figure 5A and B(Fig. 5), 0.1 % DMSO-treated A-549 and MCF-7 tumor cells used as controls showed 93-95 % viable cells (highly condensed bright green chromatin), 2-3 % viable apoptotic (bright green nucleus with condensed/fragmented chromatin), ~ 3 % late apoptotic (condensed and fragmented orange chromatin), and 1-2 % necrotic cells (non-fragmented red nucleous), respectively. After 24 h treatment, NPDO and 3-nitro-NPDO quinoidals, at their CL_50_ concentrations (Table 3(Tab. 3)), diminished the viable cells to 47-50 % (A-549) and 30-45 % (MCF-7), respectively, and increased the apoptotic cells (34-38 % (A-549) and 30-45 % (MCF-7) viable apoptotic and 11-15 % (A-549) and 9-12 % (MCF-7) nonviable apoptotic), while the percentages of the necrotic cells in both cell lines were virtually the same as those in the untreated cells, implying that the tumor cell death induced by the treatment of NPDO quinoidals occurs primarily through apoptosis.

## Concluding Remarks

The nitrogen-containing tetracyclic ortho-quinones and their nitrated derivatives serve as excellent electron-accepting and redox active substrates of single- and two-electron (hydride)-transferring electron transferring flavoenzymes CPR and NQO-1, respectively, generating reactive oxygen species, and displayed relatively high antitumor potency. The antitumor activities of nitro-quinoidals were observed to be dependent upon the position of nitro groups added to the tetracyclic system; 3- and 9-nitro-quinoidals having the highest electron-accepting potency and being virtually the most efficient enzymatic substrates, showed the highest antitumor effects while 1- and 4-nitro-quinoidals produced the least anti-tumor activities. The assessment of the electrophilic Fukui function values implies that ortho-quinone moiety of the compounds is essential for their redox action inducing cytotoxic effects; however, a relatively high local electrophilic potency defined for nitro groups of the 3- and 9-nitro-quinoidals suggests that their nitro groups might be also involved in cytotoxic action. The data obtained about the structures of (P)NPDOs, global and regional reactivities, and their antitumor activities provide some guide for synthesis of related structure compounds as antitumor agents.

## Notes

Jonas Šarlauskas and Žilvinas Anusevicius (Institute of Biochemistry, Life Sciences Center, Vilnius University, Sauletekio av. 7, Vilnius, LT-10257, Lithuania; E-mail: zilvinas.anusevicius@bchi.vu.lt) contributed equally as corresponding authors.

## Acknowledgement

The authors are highly grateful for the financial support of the Scientific Council of Lithuania (the project No. MIP-032/2014). The authors thank Audrius Laurynėnas (Vilnius University, Center of Life Science) for his excellent assistance in DFT computation. The authors also thank Lina Marčiulionytė (Lecturer at Vilnius University) for proofreading the manuscript.

## Conflict of interest

The authors declare no conflict of interest.

## Figures and Tables

**Table 1:**
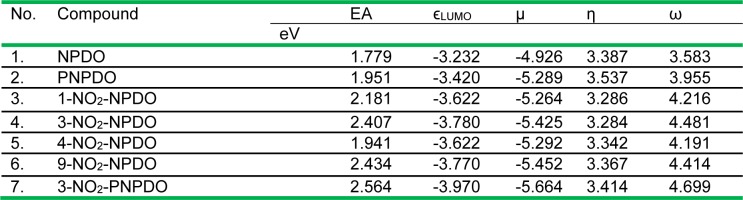
The electron affinity (EA), the LUMO energy (ϵ_LUMO_), the electronic chemical potential index (µ), the chemical hardness index (η), and the electrophilic ω index values of the ortho-quinoid compounds calculated by means of DFT-B3LYP functional with 6-31+G(d) basis set

**Table 2:**
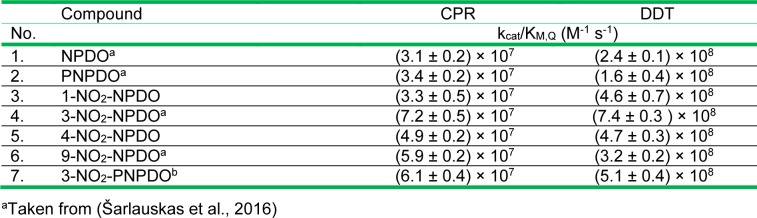
The apparent second-order rate constant (k_cat _/ K_M(Q)_) values of CPR and DT-diaphorase-mediated reduction reactions of (P)NPDO quinone compounds

**Table 3:**
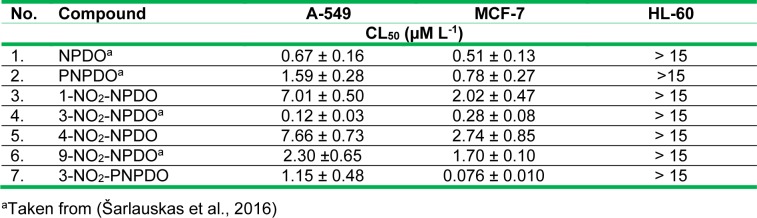
Cytotoxic effects of ortho-quinoids against A-549, MCF-7 and HL-60 cell lines (results are shown as the mean ± S.E.M.)
